# Knowledge, attitude and factors associated with induced abortion among female students ‘of Private Colleges in Ambo town, Oromia regional state, Ethiopia: a cross-sectional study

**DOI:** 10.1186/s12905-022-01935-3

**Published:** 2022-08-18

**Authors:** Rebuma Muleta Gutema, Gurmesa Daba Dina

**Affiliations:** Department of Midwifery, College of Medicine and Health Sciences, Ambo University, Ambo, Ethiopia

**Keywords:** Knowledge, Attitude, Induced abortion, Female students, Ethiopia

## Abstract

**Background:**

Around 73 million induced abortions take place worldwide each year. Six out of 10 (61%) of all unintended pregnancies, and 3 out of 10 (29%) of all pregnancies, end in induced abortion. In Africa, nearly half of all abortions occur under the least safe circumstances. In Ethiopia 35% of women obtaining induced abortions service. Therefore, thisstudy aims to assess knowledge, attitude, and associated factors towards induced abortion serviceamong female students of private Colleges in Ambo town, Ethiopia, 2022.

**Methods:**

An Institution-based cross-sectional study was conducted from January 15, 2022, to February 15, 2022, among college students in Ambo, Ethiopia. Data were collected from 631 female students using semi-structured self-administered questionnaires by a systematic sampling method. We collected data on demographics, Institutional factors: facility policy and regulation, sexual experience, knowledge, and attitude. Bivariable and multivariable logistic regression analyses were done to identify the association of dependent and independent variables using SPSS, version 26, at 95% of confidence interval by adjusting for confounding factors. Finally, variables with *p*-value ≤ 0.05 were taken as factors associated.

**Results:**

All the participants gave their responses.Among the participants 279 (44.2) have good knowledge while the majority 352 (55.8%) of the students had poor knowledge about induced abortion. Age [AOR = 4.64, 95% CI (2.95,7.30)], Marital status [AOR = 5.24, 95% CI (3.16, 8.69)], religion [AOR = 0.48, 95% CI (0.26,0.81)], Year of study [AOR = 4.51, 95% CI (2.88,7.08)], Monthly earn/income [AOR = 2.07, 95% (CI 1.40,3.07)], Ever had sex [AOR = 1.92, 95% CI (1.26,2.92)] and urban residence [AOR = 1.87, 95% CI (1.26, 4.35)] were factors associated with knowledge of students towards induced abortion. Regarding attitude, 377 (59.7%) of students had good attitude towards induced abortion. Marital status [AOR = 2.30, 95% CI (1.30, 4.0)], and Religion [AOR = 0.47, 95% CI (0.10, 2.23)] were factors significantly associated with attitude towards induced abortion.

**Conclusion:**

More than half of the participants have poor knowledge while majority of the students have a good attitude toward induced abortion. Since majority of the students (55.8%) have poor knowledge about induced abortion: health education, short course training, panel discussions and communication programs for youth on induction of abortion services is crucial.

## Introduction

Abortion is the termination of pregnancy before fetal viability(before 20 weeks of gestation according to the world health organization or before 28 weeks of gestational age (Ethiopia)), that can occur either spontaneously or induced under certain circumstances [1]. Induced abortion is the termination of pregnancy by undergoing a deliberate steps to remove or expel an embryo or fetus [2]. Induced abortion is mainly performed whenever there are some compelling reasons to end a pregnancy [3]. Unsafe abortion is defined as the termination of pregnancy by unskilled persons and/or those conducted under unhygienic conditions [4]. Lack of delivery systems, restrictive abortion laws, negative cultural and religious attitudes, and poor health infrastructure for the treatment of abortion-related complications were the main burdens of women´s health problems [5]. Each year an estimated 36- 53 million abortions are performed worldwide and from this, around 20 million abortions are considered unsafe [6]. World Health Organization estimates showed that the proportion of maternal mortality due to abortion complications ranges from 8% in Western Asia to 26% in South America, with a worldwide average of 13% [7, 8]. Even though developing countries have been applying public health approaches based on primary, secondary, and tertiary preventions in order to reduce morbidity and mortality associated with unsafe abortion, abortion still produces a deleterious effect on maternal health [9–11]. Complications of unsafe abortion cause between 50,000 and 100,000 women's deaths annually [12]. Long-term consequences like chronic pelvic pain, incontinence, obstetric problems, and infertility have been associated with complications from induced abortion [13]. In addition to seriously negative health consequences, induced abortion also has a severe economic impact, especially in poor countries with already overloaded health systems. Direct costs include skilled personnel, medications, blood for transfusion, supplies, equipment, and hospitalization costs [14, 15]. Studies in Ethiopia showedthat an estimated 620,300 induced abortions were performed in 2014 [16]. The annual abortion rate was 28 per 1,000 women aged 15–49, an increased from 22 per 1,000 in2008, and was highest especially in the urban areas (Addis Ababa, Dire Dawa, and Harari) [16]. Ethiopia has ratified laws and conventions pertaining to abortionunder certain circumstances [17]. This restrictive Law coupled with contraceptive shortages, low usage of available methods, and a high rate of sexual violence had led the country to be among the leading developing countries in abortion-related morbidity and mortality [18]. In most developing countries including Ethiopia, access to induced abortion and post-abortion care (PAC) was influenced by women’s knowledge to induced abortion services [5]. The decision as to whether to continue a pregnancy or terminate it, is fundamentally and primarily the woman’s decision, as it may shape her whole future personal life as well as family life and has a crucial impact on women’s enjoyment of other human rights [19]. A woman’s access to services is also determined in part by their positive attitude towards abortion services in order to reduce maternal mortality and morbidity [20]. Increasing access to comprehensive abortion care requires careful consideration and understanding of the multilayered physical, legal, political, economic, and cultural context of women’s daily lives. There is no assessment made on such situations in the study area. So, this study aims to assess knowledge, attitude, and factors associated toinduced abortion service among female students of Colleges in Ambo town, Oromia regional state, Ethiopia.

## Methods

A facility-based cross-sectional study was carried out among female college students in Ambo town from January 15, 2022, to February 15, 2022. Ambo town is the administrative city of West Shoa zone, and located at114 kilometers to the west of Addis Ababa the capital city of Ethiopia. According to the 2019 population estimation, the total number of residents of Ambo town was estimated to be 80,712.There are seven private educational colleges in Ambo town. The total number of students from all colleges was 10,448, of whom 5,756 were female students.

All selected non-health science female college students among the selected colleges at the time of data collection were included in the study.

### Sample size determination

The single population proportion formula was computed to get the sample size with the assumption of a 95% confidence interval, 5% margin of error, and *p* = 50%, (no previous study conducted on induced abortion), considering a 10% non-response rate and using a design effect of 1.5 yields the final sample size of 631.

### Sampling technique

To get the required sample size, stratified random sampling method was used to select study participants. First, students were stratified by their department. Then, the sample size was proportionally allocated to all the departments in the institutions based on the number of female students in each department. The sample was collected from all departments at every k^th^ interval which is obtained by dividing the total female students in the department by selected study participants.

### Variables

#### Dependent variables

- knowledge of induced abortion
- attitude of induced abortion

#### Independent variables

- Socio-demographic characteristics, Institutional factors: facility policy and regulation, sexual experience.

#### Operational definitions

Induced abortion: is the termination of pregnancy with a method recommended by world health organization, by skilled health care provider and performed under hygienic conditions.

Attitude: In this study, the attitude was used to describe the views of students toward Induced abortion.

Good attitude: respondents those answer greater than the mean attitude score is considered to have a good attitude.

Poor attitude: respondents who answered less than the mean attitude score were considered to have a poor attitude.

Knowledge: In this study knowledge was used for explaining the awareness of students about Induced abortion.

Good knowledge: The respondents who scored greater than or equal to 60% in the knowledge questions were considered as having “good knowledge”.

Poor knowledge: respondents who scored less than 60% were considered as having “poor knowledge.”

### Data collection tool, procedure and data quality control

The data were collected using a structured and self-administered questionnaire that was designed by reviewing different studies [21–24]. The questionnaire was prepared in the English version, as it is teaching/learning medium at all colleges. The questionnaire consists of variables related to socio-demographic characteristics, Institutional factors: facility policy and regulation, sexual experience, knowledge, and attitude towards induced abortion. Ten degree holder data collectors who have experience and two supervisors were recruited for the data collection. Before the actual data collection, the questionnaires were pre-tested on 5% of the total sample size ofstudents who are different from the selected institution and place. After data collection is completed, the questionnaires were checked for completeness by the investigators.

### Data analysis

The questionnaire was cleaned, coded, and entered into EPI data version 3.1. Then the data was exported to SPSS version 26 for cleaning and analysis. Frequencies, percentages, and mean were computed to describe the key variables of the study. Bivariable and multivariable analyses were used to determine the association between different factors and the outcome variables and variables having a *p*-value < 0.05 in the multivariable analysis were considered statically significant.Odds ratios and the respective 95% confidence intervals were used to assess the strength of association.

## Results

### Socio-demographic characteristics of the respondents

All of the respondents participated in this study gave their response. The mean age of the participants was 22.1 years (SD ± 2.50). One hundred ninety-seven (31.2%) of students were single, never been in a relationship. The majority of students (67.4%) reported that they were living in an urban area. Nearly, two-thirds (60.4%) of the students get 501.00–1500.00 Ethiopian birr or 10.02–30 dollars sent from family. Concerning the Year of study, 226 (35.84%) of them were attending in 1st year (Table 1).

**Table 1:** Socio-demographic characteristics of private college students in Ambo town, Ethiopia, 2022 (n = 631)

Variables	Response	Frequency	(%)
Age	< 20	139	22.0
	20–23	375	59.3
	≥ 24	117	18.5
Marital status	Never in relationship	197	31.2
	No current relationship	119	18.9
	In relationship	195	30.9
	Married	120	19.0
Religion	Orthodox	267	42.3
	Muslim	62	9.8
	Protestant	293	46.4
	Wakefata	9	1.4
Ethnic group	Oromo	563	89.2
	Amhara	64	10.1
	Tigre	4	0.6
Father's educational status	No formal education	26	4.1
	can read and write	41	6.5
	Primary education	111	17.6
	Secondary education	308	48.8
	Diploma and above	145	23.0
Mother's educational status	No formal education	78	12.4
	Primary education	227	36.0
	Secondary education	189	30.0
	Diploma and above	137	21.7
What is your father’s occupation?	Daily laborer	58	9.2
	Farmer	206	32.6
	Government employed	161	25.5
	private Employed	150	23.8
	own business	56	8.9
What is your mother’s occupation?	House wife	249	39.5
	Daily laborer	74	11.7
	private Employed	63	10.0
	Has own business	169	26.8
	Government employed	76	12.0
Residence	Urban	425	67.4
	Rural	206	32.6
Department	Accounting	175	27.7
	Economics	189	30.0
	Management	115	18.2
	Marketing management	74	11.7
	Automotive	31	4.9
	cooperative business management	47	7.4
Year of study	1st year	251	39.8
	2nd year	226	35.8
	3rd and above	154	24.4
Monthly income sends from family	< 500	77	12.2
	501.00–1500.00	381	60.4
	> 1501.00	173	27.4

#### Sexual behavior

Sexual behavior: concerning Sexual experience 366 (58.0%) of the students never had sex during the time of the interview in which most of them make an episode of sexual intercourse during the last week of the interview. Among the participants who have ever had sex (265), the majority (53.97%) of the students does not use contraceptives, stating their reason as 55 (38.46%) due to fear of side effects followed by 30 (20.97%) don’t know where to get the contraceptive. (Table 2).

**Table 2:** Sexual experience of private college students in Ambo, Ethiopia, 2022 (n = 631)

Variables		Frequency	(%)
Sexual experience	Ever had sex	265	42.0
	Never had sex	366	58.0
Most recent episode of sexual intercourse	Past week	108	40.7
	Past month	97	36.6
	More than two month back	60	22.6
Use of contraceptives during most recent coitus	Used	122	46.03
	Not used	143	53.97
Type of contraceptive used	Oral contraceptive pills	28	23.0
	Condoms	80	65.57
	Injectable	8	6.55
	Implant	6	4.91
If not used why?	I do not like them (oppose)	19	13.28
	Partner opposed	18	12.58
	Don’t know where to get them	30	20.97
	Fear of side effects	55	38.46
	Fear of the society	21	14.68
If you have any unwanted pregnancy at any time, what would you do?	To continue with the pregnancy and gave birth	161	25.5
	To terminate it	247	39.1
	I am not sure what to do	223	35.3
Have you ever had abortion(pregnancy terminated before completed 28 weeks of gestation)?	Yes	31	4.9
	No	600	95.1
where was that particular abortion induced?(for yes)	Patient’s home	4	12.9
	Abortionist’s home	2	6.4
	Hospital	14	45.16
	Health center	5	16.12
	Clinic	4	12.9
	Private health institution	2	6.4

#### Knowledge of the Respondents on Induced abortion

**Fig. 1:**
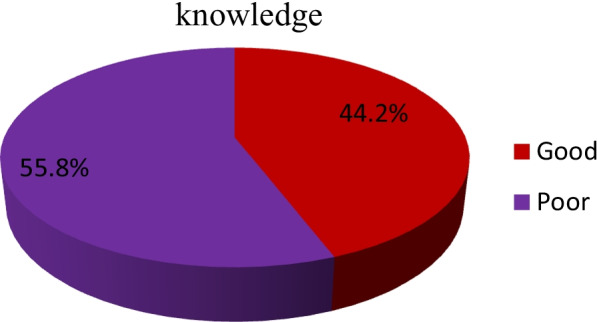
knowledge oninduced abortion among female students’ of private Colleges in Ambo town Ethiopia, 2022

Overall 279 (44.2%) students’ have good knowledge regarding induced abortion (Fig. 1).

About 297 (47.1%)) of the respondents had ever heard about induced abortion; of those 147 (49.49%) got information from health institutions. The majority (391 (62.0%)) of the respondents mentioned that the place where induced abortion was performed was in the hospital. Nearly three-fourths of 424 (67.2%) of the students believe that induced abortion services reduce the risk of women’s reproductive health problems. About 278 (44.1%) of the participants reported that induced abortion was a major health problem in Ethiopia today. More than half 403 (63.9%) of the respondents said that Ethiopia has an abortion law. Among respondents, 360 (57.1%) said below 3 months of pregnancy is the preferable time to perform induced abortion (Table 3).

**Table 3:** Knowledge regarding induced abortion among private college students in Ambo, Ethiopia, April, 2022 (n = 631)

Variables	Response	Frequency	%
Have you ever heard about the method of pregnancy termination or induced abortion?	Yes	297	47.1
	No	334	52.9
From where/whom have you ever heard about induced abortion? (n = 297)	Health institution	147	49.49
	Mass media	54	18.18
	Parents	9	3.0
	Peer/friend	87	29.29
Where do you think that induced abortion service conducted?	Hospital	391	62.0
	Health centre	48	7.6
	Private clinic	11	1.7
	Don’t know	181	28.8
Will induced abortion service reduce the risk of women’s reproductive health problem?	Yes	424	67.2
	No	28	4.4
	Don’t know	175	27.7
When is the preferable time to perform abortion?	Before 3 months of pregnancy	360	57.1
	At any time during pregnancy	89	14.1
	Don’t know	182	28.8
Is unsafe abortion a major problem, today in Ethiopia?	Yes	278	44.1
	No	170	26.9
	Don’t know	183	29.00
Ethiopia has abortion law?	Yes	403	63.9
	No	45	7.1
	Don’t know	183	29.00
For what reason is abortion legal in Ethiopia? (More than one possible)	If the pregnancy is extra-marital	49	7.7
	If pregnancy is due to rape or incest	343	54.3
	If pregnancy endangers life of the woman or fetus	232	36.76
	For woman with physical/mental disabilities	230	36.45
	For woman physically psychologically unprepared	78	12.36
	If she is financially unable to rise the child	45	7.13
	Not allowed for any reason in Ethiopia	87	13.78
What is/are some possible complication of abortion?	Heavy bleeding	111	17.6
	Uterine rupture	73	11.6
	Infertility	93	14.7
	Infections	80	12.7
	No complication	93	14.7
	Don’t know	181	28.68

#### Attitudes of respondents on induced abortion

**Fig. 2:**
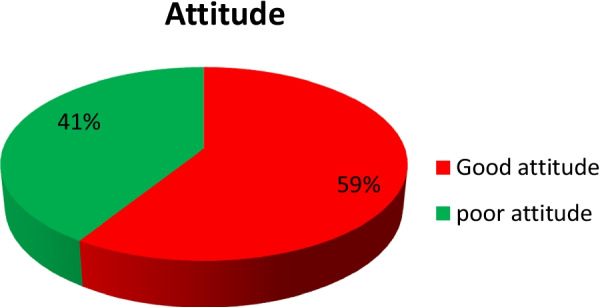
Attitude to induced abortion among female students’ of private Colleges in Ambo town Ethiopia, 2022

Among the study participants, 377 (59.7%) had a good attitude toward induced abortion (Fig. 2).

The majority 584 (92.6%) of the respondents agreed that Induced abortion should be fully legalized and accessible in Ethiopia. The majority (461 (72.8%)) of respondents have disagreed with the statement which says the outcome of abortion done in health institutions and by traditional practitioners is similar. More than two-thirds of 454 (71.9%) students agreed that induced abortion has no complications. The finding also showed that 570 (90.3%) of the respondents agreed that pregnant women should decide to abort or not, not their family or clinician (Table 4).

**Table 4:** Attitude towards induced abortion among private college students in Ambo town, 2022 (n = 631)

Variables	Disagree n (%)	Neutral n (%)	Agree n (%)
If your sister, relative or friend encounter unwanted pregnancy, it is good if advised on induced abortion	4 (0.6)	27 (4.3)	602 (95.4)
Induced abortion should be recommended to every woman with unwanted pregnancy	41 (6.5)	27 (4.3)	563 (89.2)
Induced abortion should be fully legalized and accessibleinEthiopia	28 (4.4)	19 (3.0)	584 (92.6)
Induced abortion has no complication	102 (16.2)	75 (11.9)	454 (71.9)
Induced abortion can kill because of its complications	486 (77.0)	59 (9.4)	86 (13.6)
Abortion should be done every where	560 (88.7)	44 (7.0)	27 (4.3)
The outcome of abortion done in health institution and by traditional practitioners is almost similar	596 (94.5)	23 (3.6)	12 (1.9)
All women who undertake induced abortion will suffer negative mental health effects	447 (70.8)	78 (12.36)	104 (16.48)
Contraceptive cannot prevent unwanted pregnancy	544 (86.2)	79 (12.5)	8 (1.3)
It is Pregnant women whoshould decide to abort or not but not her family or clinicians	37 (5.9)	24 (3.8)	570 (90.3)

### Factors Affecting Students’ Knowledge towards Induced abortion

In the bivariate and multivariate analysis, the factors that found to have an association with knowledge towards induced abortion among private college female students were age, marital status, religion, residence, year of study, monthly earn and Sexual experience. Students found in the age group of 21-23and ≥ 24 were 4.64 times and 2.12 times more likely to have good knowledge of induced abortion than those who are in the age group of < 20 [AOR = 4.64, 95% CI (2.95, 7.30)], [AOR = 2.12, 95% CI (1.35, 3.32)] respectively. Students who are Single, with no current relationship, in a relationship, and Married were 5.24times, 2.48 and 1.99 times more likely to have good knowledge of induced abortion than those who are Single, never in a relationship [AOR = 5.24, 95% CI (3.16, 8.69)], [AOR = 2.48, 95% CI (1.43, 4.31)] and [AOR = 1.99, 95% CI (1.23, 3.23)] respectively. Regarding religion Muslim respondents were 0.48 times less likely knowledgeable than Orthodox [AOR = 0.48, 95% CI (0.26, 0.81)]. The respondents who came from the urban area were 1.87times more knowledgeable than those living in rural areas [AOR = 1.87, 95% CI (1.31, 2.80)]. Students who attend 2nd year and 3rd and above were 4.51times and 2.05 times more likely to have good knowledge of induced abortion than 1st-year students [AOR = 4.51, 95% CI (2.88, 7.08)], [AOR = 2.05, 95% CI (1.31, 3.20)] respectively. Respondents whommonthly income sent from family > 1500.00 EB/30 dollars were 2.07times more likely to have good knowledge than those whommonthly income sends from family < 500.00 EB/10.02 dollars [AOR = 2.07, 95% CI (1.40, 3.07)]. Students who ever had sexwere1.92times more likely to have good knowledge about induced abortion than those who never had sex [AOR = 1.92, 95% CI (1.26, 2.92)] (Table 5).

**Table 5:** Bivariable and Multivariable analysis of factors associated with knowledge of induced abortion service among female students in Ambo, Ethiopia, 2022 (n = 631)

Variables		Knowledge level		COR (95%CI)	AOR (95%CI)	*p*-value
		Good n (%)	Poor n (%)			
		279 (44.2)	352 (55.8)			
Age	< 20	43 (15.5)	96 (27.3)	1	1	
	21–23	159 (57.2)	215 (61.1)	4.49 (2.85,7.08)	4.64 (2.95,7.30)*	0.002
	≥ 24	76 (27.3)	41 (11.6)	2.14 (1.36,3.36)	2.12 (1.35,3.32)*	0.011
Marital status	Never in relationship	55 (19.7)	142 (40.3)	1	1	
	No current relationship	50 (17.9)	69 (19.6)	5.14 (3.02,8.72)	5.24 (3.16, 8.69)*	0.004
	In relationship	96 (34.4)	99 (28.1)	2.52 (1.44, 4.42)	2.48 (1.43, 4.31)*	0.009
	Married	78 (28.0)	42 (11.9)	1.38 (0.83, 2.32)	1.99 (1.23, 3.23)*	0.012
Religion	Orthodox	101 (36.2)	166 (47.2)	1	1	
	Muslim	37 (13.3)	25 (7.1)	0.41 (0.23,0.72)	0.48 (0.26,0.8)*	0.008
	Protestant	136 (48.7)	157 (44.6)	0.69 (0.49,0.8)	0.86 (0.60,1.24)	
	Wakefata	5 (1.8)	4 (1.1)	0.48 (0.12,1.8)	0.40 (0.10,1.6)	
Father's educational status	No formal education	12 (4.3)	14 (4.0)	1	1	
	can read and write	11 (3.9)	30 (8.5)	1.18 (0.51,2.73)	0.82 (0.33,2.00)	
	Primary education	43 (15.4)	68 (19.3)	2.76 (1.28,5.93)	1.99 (0.88,4.49)	
	Secondary education	140 (50.2)	168 (47.7)	1.60 (0.97,2.64)	1.18 (0.67, 2.07)	
	Diploma and above	73 (26.2)	72 (20.5)	1.21 (0.81, 1.80)	1.06 (0.70, 1.61)	
Mother’s occupation	House wife	92 (33.0)	157 (44.6)	1	1	
	Daily laborer	31 (11.1)	43 (12.2)	1.70 (1.01,2.86)	1.54 (0.87, 2.71)	
	private Employed	29 (10.4)	34 (9.7)	1.38 (0.72,2.64)	1.25 (0.64, 2.45)	
	Has private business	89 (31.9)	80 (22.7)	1.17 (0.60,2.21)	1.11 (0.56, 2.20)	
	Government employed	38 (13.6)	38 (10.8)	0.89 (0.52,1.54)	0.88 (0.51,1.5)	
Residence	Rural	210 (75.3)	215 (61.1)	1	1	
	Urban	69 (24.7)	137 (38.9)	1.86 (1.29, 2.69)	1.87 (1.31, 2.80)*	0.002
Year of study	1st year	74 (26.5)	177 (50.3)	1	1	
	2nd year	105 (37.6)	121 (34.4)	4.49 (2.85,7.03)	4.51 (2.88,7.08)*	0.001
	3rd and above	100 (35.8)	54 (15.3)	2.14 (1.36,3.36)	2.05 (1.31,3.20)*	0.006
Monthly income sends from family	< 500.00	35 (12.5)	42 (11.9	1	1	
	501.00–1500.00	150 (53.8)	231 (65.6)	1.42 (0.83, 2.44)	1.64 (0.92,2.92)	
	> 1500.00	94 (33.7)	79 (22.4)	1.83 (1.27,2.63)	2.07 (1.40,3.07)*	0.004
Sexual experience	Ever had sex	158 (56.6)	107 (30.4)	1.95 (1.29,2.96)	1.92 (1.26,2.92)*	0.007
	Never had sex	121 (43.4)	245 (69.6)	1	1	

^*^Associated variables with *p*-value < 0.05

Factors Affecting Students’ Attitude towards Induced abortion:religion and marital status had an association with the attitude towards induced abortion in the bivariable and multivariable logistic regression analysis. Students who are in a relationship were 2.30 times more likely to have a good attitude towards induced abortion than those who are never in a relationship [AOR = 2.30, 95% CI (1.30, 4.0)]. Regarding religion Protestant respondents were 0.48 times less likely have good attitude than Orthodox [AOR = 0.48, 95% CI (0.10, 2.01)](Table 6).

**Table 6:** Bivariable and Multivariable analysis of factors associated with attitude of induced abortion among female students in Ambo, Ethiopia, 2022 (n = 631)

Variables		Attitude		COR(95%CI)	AOR(95%CI)	*p*-value
		Good n (%)	Poor n (%)			
		377 (59.7)	254 (40.3)			
Age	< 20	83 (22)	56 (22)	1	1	
	21–23	215 (57.2)	159 (62.6)	1.37 (0.71, 2.67)	1.27 (0.76, 2.12)	
	≥ 24	78 (20)	39 (15.4)	1.30 (0.77,2.13)	1.43 (0.92,2.21)	
Marital status	Never in relationship	120 (31.8)	77 (30.3)	1	1	
	No current relationship	60 (15.9)	59 (23.2)	1.15 (0.72, 1.84)	1.41 (0.78, 2.55)	
	In relationship	119 (31.6)	76 (29.9)	2.07 (1.23,3.41)	2.30 (1.30,4.01)*	0.005
	Married	78 (20.7)	42 (16.5)	1.18 (0.74, 1.88)	1.32 (0.79, 2.22)	
Religion	Orthodox	164 (43.5)	103 (40.6)	1	1	
	Muslim	40 (10.6)	22 (8.7)	0.38 (0.53,0.31)	0.57 (0.14,2.34)	
	Protestant	168 (44.6)	125 (49.2)	0.29 (0.45, 0.10)	0.47 (0.10,2.01)	
	Wakefata	5 (1.3)	4 (1.6)	0.55 (0.65, 0.16)	0.70 (0.17,2.81)	
Residence	Rural	260 (69.0)	165 (65.0)	1	1	
	Urban	117 (31.0)	89 (35.0)	0.82 (0.57,1.16)	0.80 (0.57,1.14)	
Year of study	1^st^ year	135 (35.8)	116 (45.7)	1	1	
	2^nd^ year	148 (39.3)	78 (30.7)	1.29 (0.85,1.96)	1.28 (0.85, 1.94)	
	3^rd^ and above	94 (24.9)	60 (23.6)	0.82 (0.53,1.28)	0.82 (0.53,1.26)	
Monthly earn	< 500.00	49 (13.0)	28 (11.0)	1	1	
	501.00–1500.00	227 (60.2)	154 (60.6)	0.67 (0.38,1.180	0.68 (0.38,1.19)	
	> 1500.00	101 (26.8)	72 (28.3)	0.93 (0.64,1.34)	0.95 (0.65,1.37)	
Sexual experience	Ever had sex	156 (41.4)	109 (42.9)	1.51 (0.96, 1.98)	1.89 (1.64,2.09)*	0.008
	Never had sex	221 (58.6)	145 (57.1)	1	1	

^*^Associated variables with *p*-value < 0.05

## Discussion

This study revealed that 44.2% of the students have good knowledge about induced abortion. This finding is consistent with the study done at Mekelle University, Northern Ethiopia (44.1%), higher than studies conducted in India (36%), and WolaitaSodo University, Ethiopia (38.8%). This might be due to the differences in the year of study participants, in our study, the study participants included were the first year and above and socioeconomic status. This result is lower than the study conducted in Kampala, Uganda (72.4%) and in Gondar City, Northwest Ethiopia (68.4%)respectively [21, 22, 25–27]. The reason for this discrepancy might be due to differences in study participants' educational level, exposure to sources of information (mass media, radio, television, etc.), and geographical location (urban or rural). Regarding age, students found in the age group of 21–23 and ≥ 24 were five times and two times more likely to have good knowledge of induced abortion than those who are in the age group of < 20. The probable justification might be the fact that as age increases; students’ exposure to information and education regarding induced abortion could also increase. This finding is in line with a cross-sectional study conducted in Kampala Uganda and in Gondar, Northwest Ethiopia [21, 27]. Students who are currently not in a relationship, in a relationship, and Married were five times, two times, and nearly two times more likely knowledgeable about induced abortion than those who have been never in a relationship respectively. This may be because the chance of females who are in relation to be exposed to sexual intercourse and getting unwanted pregnancy is high, their knowledge of the solution also increase.This result is in line with the study done in Buenos Aires, Argentina, and South Africa [28, 29]. Regarding religion, Muslim respondents were found to be less knowledgeable about induced abortion than Orthodox Christians. This is supported by the study done in South Africa [29]. This may be due to different religions having a different point of view related to abortion [30]. This study also showed that students who came from the urban area were nearly two times more knowledgeable than those living in rural areas. This is in line with the study done in Gondar, Ethiopia [21]. Students who attend2^nd^and 3^rd^and above years of study were four times and two times more likely to have good knowledge of induced abortion than 1st-year students respectively. This finding was supported by studies done at the University of Buenos Aires, Argentina, and kebribayah town in the Somali region, Ethiopia [23, 28]. This is due to the fact that as the year of study increases, the level of knowledge of students also increases [31]. Respondents whom monthly income sent from family > 1500.00 EB/30 dollars were two times more likely to have good knowledge than those whommonthly income sent from family < 500.00 EB/10.02 dollars. The justification for this might be because participants who have good income get access to information through media, education, and peer education, which may lead to having provided knowledge. Participants who ever had sex were nearly two times more likely to have good knowledge about induced abortion than those who never had sex. This finding is in agreement with a study done in South Africa [29]. Concerning the attitude, this study shows that 59.7% of the students have a good attitude toward induced abortion. This result is nearly in line with the studies done in Gondar City, Northwest Ethiopia (57.0%), and in Mekelle University, Ethiopia respectively(52.8%) [21, 22]. However, this result is lower than other cross-sectional studies conducted at Mizan-Tepi University (74.17%) and South Africa (70%), respectively [29, 32]. This difference might be due to the study participants, and socioeconomic statusamong the participants.This result is higher than a study conducted in the Somali Region, Ethiopia, which revealed 40.7% of students had a favorable attitude toward induced abortion [23]. The reason for this variation may be due to cultural beliefs, religious points of view, and socioeconomic status. Students who are in a relationship were nearly two times more likely to have a good attitude towards induced abortion than those who are never in a relationship. This result is supported by a cross-sectional study conducted in Gondar City [21]. Participants who ever had sex were nearly two times more likely to have a good attitude towards induced abortion than those who never had sex. The explanation would be due to abortion experience may be compounded by pregnancy due to sexual abuse or transactional sex.

## Conclusion

Though significant progress has been achieved, knowledge and attitude towards induced abortion remain inadequate and substandard in the study area. This could be attributable to socioeconomic status, cultural beliefs, and the problem of information dissemination. School health programs have to be considered and redesigned and attention must be given to the students on reproductive health issues. Governments as well as non-government organizations along with the most respectable community elders and religious leaders should work on these issues to develop positive knowledge and a good attitude toward induced abortion. Forums and panel discussions on induced abortion need to be undertaken, especially among youths and students who come from rural areas. To prevent maternal mortality and morbidity which results from the complication of unsafe abortion, youth-friendly service has to be expanded to the rural part of Ethiopia.

Some limitations of this study: The social desirability bias due to the nature of the sensitivity issues of abortion and the past history of abortion might be affected by recall bias.

## Data Availability

The datasets generated and/or analyzed during the current study are available from the corresponding author on reasonable request.
